# Determinants of risky sexual practice, drug abuse and alcohol consumption in adolescents in Iran: a systematic literature review

**DOI:** 10.1186/s12978-019-0779-5

**Published:** 2019-07-24

**Authors:** Vahid Yazdi-Feyzabadi, Mohammad Hossein Mehrolhassani, Farzaneh Zolala, AliAkbar Haghdoost, Nadia Oroomiei

**Affiliations:** 1 PhD in Health Policy, Health Services Management Research Center, Institute for Futures Studies in Health, Kerman University of Medical Sciences, Kerman, Iran; 2 PhD in Health Services Management, Medical Informatics Research Center, Institute for Futures Studies in Health, Kerman University of Medical Sciences, Kerman, Iran; 3 Ph.D. in Epidemiology, Social Determinants of Health Research Center, Institute for Futures Studies in Health, Kerman University of Medical Sciences, Kerman, Iran; 4 PhD in Epidemiology, Health Modeling Research Center, Institute for Futures Studies in Health, Kerman University of Medical Sciences, Kerman, Iran; 5 PhD Candidate in Health Policy, Faculty of Management and Medical Informatics, Kerman University of Medical Sciences, Medical University Campus, Haft-Bagh Highway, Kerman, 7616913555 Iran

**Keywords:** Determinants, Risky sexual behavior, Synthetic drugs, Drug abuse, Alcohol consumption, Adolescents, Iran

## Abstract

**Background:**

Evidence shows that the prevalence of risky sexual practice, drug abuse, and alcohol consumption behaviors in low and middle income countries such as Iran is not in a favorable condition. Preventive programs against these behaviors in Iran are very rare, and the results are unclear, which may be due to the lack of deeply and systematically understanding of the determinants of these behaviors. Evidence suggests that these behaviors are coincidence. So all of these behaviors were examined together. The present study was conducted aiming at determining the reasons for the occurrence of these behaviors among 15–19-year-old adolescents in Iran.

**Methods:**

The Preferred Reporting Items for Systematic Reviews and Meta-analyses guidelines were followed to review published and unpublished studies in Iran. The databases used were Scopus, PubMed, Web of Science, and Cochrane Library. The query terms were “Synthetic Drug” OR “Designer Drug”, AND Adolescents OR Teenagers OR Juvenile, AND Iran. The Joanna Briggs Institute Critical Appraisal Checklist was employed for critical appraisal. The quantitative studies using the regression model to analyze the factors affecting these behaviors were studied as the form of the theme. For analyzing the data, narrative synthesis and thematic analysis were used.

**Results:**

Twelve studies were meticulously reviewed. The findings were classified into five main themes (including individual, family, friends, school, and community) and 26 sub-themes. The most frequent main theme and sub-themes were respectively Family, Higher age, Male gender, Weak religious beliefs, Low self-esteem, Anti-social behaviors in family, Mother’s employment, Parenting style, Poor intimacy of parents, Absence of parents, Peer pressure, and Lack of appropriate recreation. No primary study has referred to the political, economic, or policy factors affecting such behaviors.

**Conclusions:**

The most identified sub-themes belong to family factors. Iran is a country with ideology of Islam; however, being Muslim does not guarantee adherence to all Islamic guidelines. So being Muslim is not a good reason to prevent these behaviors. Iran needs precise policy making in this area through considering family structure. It is also suggested that primary studies referring to the political, economic, or policy factors affecting such behaviors should be carried out.

## Plain English summary

Iran is a developing country having a republic and Islamic government. While out-of-norm communications with the opposite gender, any sexual contact between a girl and a boy outside official marriage frameworks, alcohol abuse, and drug abuse are not accepted in this country, the prevalence of these behaviors is not in a favorable condition in Iran. Preventive programs against these behaviors in Iran are very rare, and the results are unclear which may be due to the lack of deeply understanding the determinants of these behaviors. Therefore, this systematic review was conducted aiming at determining the reasons for the occurrence of these behaviors among 15–19-year-old adolescents in Iran. In general, 12 articles were reviewed and it was found that the probability of occurrence of these behaviors in males is more than that in females. The likelihood of occurrence of these behaviors increases in late adolescence. Strong religious beliefs are a preventative factor. When adolescents suffer from lack of confidence and feeling badly about their self, the probability of occurrence increases. Poor intimacy of parents, absence of parents, and spending little time with teenagers will increase the probability of occurrence. Although parents want their children to be well-behaved and successful, their strict manner of parenting does not allow for much flexibility. Moreover, when parents make very few demands of their children and they are often indifferent, dismissive, or even completely neglectful, the likelihood of occurrence of these behaviors increases. Therefore, Iran needs precise policy making in this area through considering family structure.

## Background

Life cycle provides an important perspective for public health measures. Events in one stage of life will affect the whole life. Therefore, what happens during the early years of life may affect the health and development of adolescents, and the health status of adolescents will also affect their adulthood health and ultimately the health of the next generation [1]. Having been known as an age range of 10 to 19 years and the transition from childhood to adulthood, adolescence is a period of rapid physical, mental, social, and cultural development as well as cognitive changes, and is associated with striving to confront and overcome challenges and creating a sense of identity and independence. It generally forms the basis of adults and elderlies’ lives in the future. Effective interventions in adolescence may protect the public health investments in childhood [2, 3].

High-risk behaviors are defined as behaviors that potentially put people at risk of significant harm and impede the prosperity of their talents. High-risk behaviors in adolescence can endanger the individuals’ health. One of these behaviors is high-risk sexual behavior that can have consequences such as unwanted pregnancy, multiple sex partners, inadequate use of condoms, HIV infection, and other sexually transmitted diseases [4]. Furthermore, regarding the relationship between low maternal age and increased newborn mortality, the reduction of pregnancy in adolescents is the core of reducing maternal mortality and improving newborn survival [5]. Evidence suggests that this behavior is typically associated with other high-risk behaviors such as drug abuse particularly synthetic drug abuse in adolescents, and alcohol consumption. Coincidence of these behaviors can increase health problems among adolescents [6].

Drug and alcohol abuse can lead to poor brain performance and lack of concentration, school escape, bad grades, agonistical relationships with family, conflicts with the judiciary and the police in adolescents and may also lead to drug addiction in adulthood [7, 8]. These behaviors can exacerbate high-risk sexual behaviors [9]. A study by Clayton et al. showed that the risk of engaging in high-risk sexual behaviors among students who used synthetic drugs was higher than that of other students [10].

The population of adolescents in low and middle income countries is significant. Given the social, economic and cultural conditions of these countries, the harm caused by high-risk behaviors of this group of people is more significant compared to high income countries. Although adolescent pregnancies are one of the most important causes of maternal and child mortality and also the continuation of the intergenerational health-sickness-poverty cycle, about 16 million adolescent girls (15–19 years old) annually give birth to children in these countries [11, 12]. Pregnancy of teenage girls can also have negative social and economic effects on these girls, their families and their communities. Single pregnant teens may face threats, violence, stigma and rejection from parents, peers and the community. Moreover, due to the conditions of education and training in these countries, they may have to leave school. Therefore, given their lack of sufficient skills and knowledge in the future, they will be unable to find suitable jobs and become more involved with economic problems. All these probabilistic problems may increase alcohol and drug abuse among these individuals, and consequently, governments will face more problems in these countries. On the other hand, the misuse of drugs and alcohol increases the probability of having risky sexual behaviors. Therefore, in order to maintain the health of society, improving social and economic conditions, paying attention to adolescent health and doing proper policy-making in this area are some of the main duties of communities. To do policy-making in this area, it is first necessary to identify the factors influencing these behaviors and understand them more deeply [13–15].

Iran as a middle income country having a republic and Islamic government has a significant teenage population [16]. In the Iranian society, religious values, cultural norms, and traditional practices emphasize the strength of family structure, obedience of adolescents from their parents, respect for them, and observation of Islamic sects. Out-of-norm communications with the opposite gender and any sexual contact between a girl and a boy outside the family and official marriage frameworks, alcohol abuse, and drug abuse are not accepted in this country. Sale and consumption of alcohol and drugs are prohibited in Iran. However, like in other developing countries, it seems that these traditional attitudes and cultural values have changed in recent years [17]. The prevalence of risky sexual behaviors, the use of drugs especially synthetic drugs, the consumption of alcohol as risky behaviors has increased in recent years in Iran [18]. The outcomes of sexual behaviors outside family frameworks include unwanted pregnancies, sexually transmitted diseases, and other consequences mentioned in various studies in the world. Furthermore, according to the studies conducted in Iran, these behaviors cause anxiety, depression, disclosure of adolescent sexual relationships, dishonor, fight and divorce of the parents blaming each other for adolescent behavior, depriving the adolescents from social activities such as continuing education and being rejected. On the other hand, considering the prohibition of alcohol and drug use in Iran, users are hidden groups that will not be identified until they seriously harm their own health and community [19].

Although the number of studies conducted regarding these behaviors in Iranian adolescents has been significant in recent years, the interventions and preventive programs against these behaviors in Iran are very rare, and the results of these interventions are unclear, and there is no evaluation of the results [20] which may be due to the lack of deeply and systematically understanding of the factors affecting high-risk behaviors. Hence, identifying these factors based on valid and reliable evidence can contribute to designing and implementing appropriate structural interventions to prevent and control these behaviors [19, 21]. Therefore, this systematic review and narrative synthesis was carried out aiming at determining the reasons for the occurrence of risky sexual behaviors, synthetic drug abuse, and alcohol consumption among 15–19-year-old adolescents in Iran.

## Methods

### Search strategy and study selection

This systematic literature review was guided by the Preferred Reporting Items for Systematic Reviews and Meta-analyses (PRISMA) statement [22]. To identify the studies relevant to the issue, international databases (including Scopus, PubMed, Web of Science, Cochrane Library, and Embase) were searched for peer-reviewed published articles. Moreover, to prevent reporting the research results only from published materials, the grey literature was also searched. To access unpublished information sources, we first contacted some experts in the Ministry of Health and Medical Universities and asked them to provide us with relevant information, if any. All unpublished and published studies and peer reviewed articles from Iran’s revolution (1979) up to March 30, 2018 were included in this review.

Studies have been ongoing since 2006, because for many years this area has been neglected in Iran and has been slowly taken into consideration since 2006, but the volume of articles was still low until the volume of these articles increased in 2011 and later [23].

The search strategy was mainly the systematic use of English keywords with all possible combinations. To improve the search sensitivity, general keywords were used as follows: “Synthetic Drug” OR “Designer Drug” OR “Customized Drug”, AND Adolescents OR Teenagers OR Juvenile, AND Iran. Additionally, the reference list of the published studies was evaluated to increase sensitivity and select further studies. Search evaluation was randomly conducted by an independent researcher (MH..M), and it was confirmed that no studies were excluded.

### Data extraction and quality assessment

Given that adolescence period is divided into two periods, and risk taking behaviors increase in the second period (15–19 years), the likelihood of occurrence of these behaviors increases [24, 25]. So the present study aimed to determine the reasons for the occurrence of risky sexual behaviors, consumption of synthetic drugs and alcohol in adolescents aged 15 to 19 years. Inclusion and exclusion criteria were shown in Table 1.

**Table 1:** Inclusion and exclusion criteria

Inclusion criteria	Exclusion Criteria
Populations	
Iranian teens aged 15 to 19 years old	Children, Youth, Teenagers under 15 years old
Reported Findings	
Published and peer reviewed articles, reports with content of the causes and the factors affecting high-risk sexual behaviors, drug use, and alcohol consumption.	Reports, studies, and theses on the prevalence Reports, studies, and theses with content of the causes and factors affecting other high-risk behaviors Reports, studies, and theses with a clinical approach Letter to the editors, Editorial articles, Commentary articles
Study Design	
Published and unpublished, mixed methods, or quantitative studies, reports, theses	Websites, blogs, anecdotal evidence
Countries, dates, language	
Iran From 1979 up to March 30, 2018 Studies reported in English	Other countries
	Other languages

Quantitative studies using the regression model to analyze the factors affecting these behaviours were also studied as the form of the theme. [26]

**Fig. 1:**
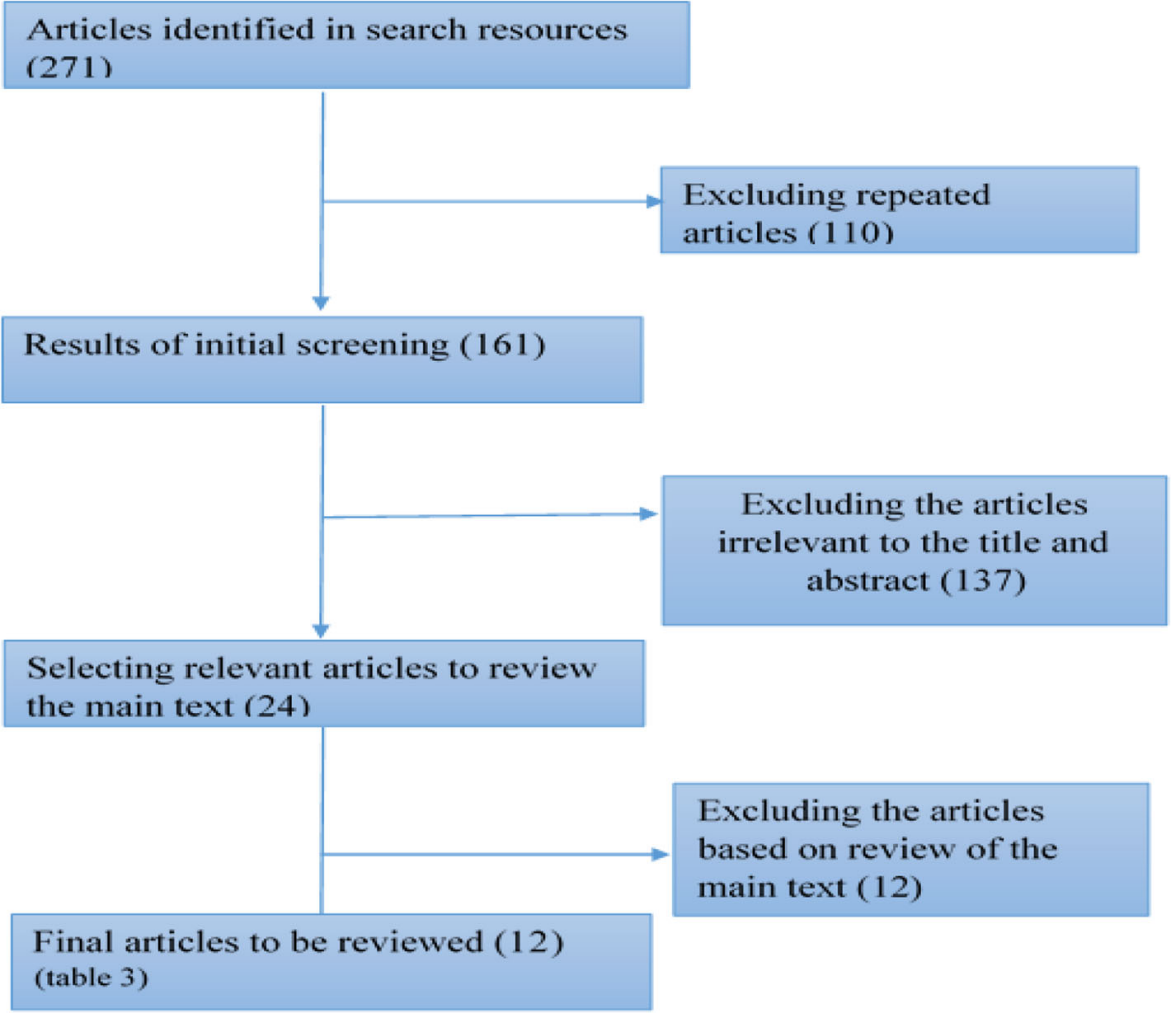
Process of study selection

The investigation of the findings to identify and eliminate repetitive research was conducted in a way that prevented the bias (Fig. 1). In order to avoid selection bias, the process of selecting the studies was independently conducted by two researchers (MH.M and N.O); contradictions were also assessed by a third researcher before a decision was made to either remove or select each study for further analysis. Furthermore, in order to avoid publication bias, duplicate articles were omitted. Moreover, grey literature were reviewed but they were irrelevant to the title and abstract so that they were excluded from the study.

The quality of the included literature was independently assessed by two reviewers (VYF and N.O). The Joanna Briggs Institute Critical Appraisal Checklist was used for critical appraisal. This research instrument contained a separate appraisal checklist for each type of study design. For each question of the checklist, a score was considered. Studies with quality assessment score more than 50% were included in the final review. For example, if the checklist had 10 questions, if the article had a minimum score of 6 out of 10, it would have entered the study.

Any disagreement between the reviewers was resolved through discussion, and by involving a third reviewer.

### Data analysis

Finally, each of the selected studies (Table 2) was meticulously read to identify key concepts and themes. The assessment of all extracted data by analyzing the final studies was conducted through narrative synthesis and thematic analysis identifying the prominent themes emerging from the evidence [27, 28]. After completing the table of data extraction, two members of the team coded the data separately (N.O, MH.M). The themes were reviewed by the third person in the team (VYF). Disagreements in coding and classification were resolved by holding a meeting with all the research team members, and an agreement was reached.

**Table 2:** A summary of characteristics of included studies

First Author	Year	Language	Study design	Setting	Data collection tools	Sampling method
Mahmoodabad SS	2017	English	Quantitative^a^	High school teens of Isfahan	questionnaire	Cluster
Bahraei A	2017	English	Quantitative^a^	Teens aged 15–18 in Tehran	questionnaire	Cluster
Haghdoost AA	2014	English	Quantitative^a^	Kerman(high and pre-university schools)	questionnaire	Stratified
Nazerzadeh M	2014	English	Quantitative^a^	Teens in Ilam (students of grade 10)	questionnaire	stratified
Panahi R	2014	English	Quantitative^a^	Teenager aged 15–19 in Kermanshah	questionnaire	Cluster
Bahraei A	2013	English	Quantitative^a^	Teens aged 15–18 in Tehran	questionnaire	Cluster
Khalajabadi F	2012	English	Quantitative^b^	Different districts of Tehran	questionnaire	cluster
Khoshabi K	2010	English	Quantitative^b^	High school teens of Tehran	questionnaire	Cluster
Farhadinasab A	2008	English	Quantitative^a^	Adolescents in Hamedan city	questionnaire	snowball
Mohammad Poursal A	2007	English	Quantitative^a^	Grade-11 boys in Tabriz	questionnaire	cluster
Kazem M	2007	English	Quantitative^a^	Tehran (teenage boys aged 15–18)	questionnaire	cluster
Mohammadi MR	2006	English	Quantitative^a^	Teenage boys aged 15–18 in Tehran	questionnaire	cluster

^a^Cross-sectional

^b^Survey

## Results

Twelve studies were meticulously reviewed. No primary study referred to the political, economic, or policy factors affecting such behaviors. The findings were classified into five main themes including individual, family, friends, school and community factors and 26 sub-themes. (Table 3).

**Table 3:** Findings from articles review

Themes	subtheme
Individual	Higher age [59, 60] Male gender [51, 59, 61–63] Weak religious beliefs [45, 64, 65] Low risk perception [66, 67] Low self-esteem [57, 63] Satisfying Curiosity [64] Feeling Pleasure [36]
Family	parenting style [59] poor intimacy of parents [59] Greater amount of pocket money [65] Anti-social behaviors in family [51, 59, 61, 68, 69] Family positive attitude to anti-social behaviors [59] Family income [60, 70] Satellite access [57] mother’s employment [51, 59, 65] Insecure attachment [51] Number of siblings [45, 59] Absence of parents [57]
Friends	Peer pressure [61, 64] Friends with risky behaviors [64]
School	Academic failure [64] Failure to respond to social needs [64]
Community	Social disorder [64] Lack of appropriate recreation [64] Easy access to drugs and alcohol + [64] Access to the Internet [57]

+only in articles about alcohol and drugs

‘Individual factors’ means the characteristics of individuals that affect occurrence of these behaviors. Individual factors theme includes seven sub-themes. The findings showed that higher age, male gender, weak religious beliefs, low risk perception, low self-esteem, satisfying curiosity, and feeling pleasure increase the likelihood of these behaviors.

When adolescents grow older and move toward the end of the adolescence, the likelihood of these behaviors increases. Also male gender is a biological factor increasing the likelihood of occurrence of these behaviors. Strong religious beliefs can be a deterrent to these behaviors. Therefore, in teenagers with poor religious beliefs, the likelihood of these behaviors increases.

The findings demonstrated that many teenagers showed such behaviors to satisfy their curiosity, feeling of pleasure, fulfill self-esteem, avoidance of problems, feeling self-greatness and power. Moreover, the likelihood of these behaviors was greater in adolescents perceiving little risk of engaging in these behaviors.

Family factors theme includes 11 sub-themes. The findings showed that autocratic parenting styles and negligent parenting style, poor intimacy of parents, greater amount of pocket money, anti-social behaviors in family, family positive attitude to anti-social behaviors, family income, satellite access, mother’s employment, insecure attachment, low siblings, and absence of parents increase the likelihood of these behaviors.

The probability of the incidence of such behaviors increases in parents who do not establish an intimate relationship with their teenagers, and the teenagers do not feel protected by the family, are constantly targeted by unjust criticism by family and are subject to violence, have in fact autocratic parenting styles, and the probability of the incidence of such behaviors increases. On the other hand, families who choose a negligent parenting style allow their children to do whatever they like, and impose few limitations on them are at risk of these behaviors. Family conflicts and unhealthy emotional relationships between parents, and the absence of both parents in adolescent life for different reasons such as parental divorce, absence of father, and living with one parent were risk factors. The findings also showed that the prevalence of these behaviors was higher in families with a family history of antisocial behaviors, or a positive attitude toward such behaviors. Another factor is family income. Both high and low incomes are referred to as factor risk in studies.

The likelihood of high-risk behaviors in adolescents may increase due to the increase in teenage pocket money. Access to satellite was another reason for the occurrence of such behaviors. The increase in mother’s employment was another issue. The low siblings is another sub-theme.

Friends factors theme includes two sub-themes. The findings showed that peer pressure and having friends with high-risk behaviors increase the likelihood of these behaviors. Adolescents follow their friends since feeling the need for belonging to a friend’s group increases at this age. If these friends are involved with high-risk behaviors, the possibility of the occurrence of these behaviors in adolescents will increase through following friends and peer pressure.

School factors theme includes two sub-themes. The findings showed that academic failure of students and failure of school structure in response to social needs increase the likelihood of these behaviors. In fact, schools affect high-risk behaviors through the status of adolescents at school and the overall school structure.

Community factors theme includes four sub-themes. The findings showed that access to the internet, social disorder, lack of appropriate recreation, and easy access to drugs and alcohol increase the likelihood of these behaviors.

## Discussion

Findings of the studies indicated that these behaviors were not one-dimensional and were due to the impact of individual, family and environmental factors. One of the influential factors is the characteristics of individuals. Male gender as a biological factor and being in late adolescence age increase the likelihood of these behaviors. Muche et al. stated that sexual relationships increase at the end of the adolescence [29]. The ‘weak religious beliefs’ is another influential factor. In their study, Seghatoleslam et al. claimed that these behaviors are prohibited in Islamic sects, and families with strong religious beliefs are less likely to commit such behaviors [30]. Iran is a country with ideology of Islam; however, being Muslim does not guarantee adherence to all Islamic guidelines [31]. So being Muslim is not a good reason to prevent these behaviors.

The next risk factor is perceiving little risk of engaging in these behaviors. Considering increase of early puberty among Iranian adolescents, the interval between sexual maturity and slow rational evolution of the cognitive control system could lead to risk-taking behaviors in adolescents [32].

Moreover, teenagers whose need for self-esteem is not fulfilled in their family, school, and community structures may need to perform such behaviors to adopt their own feelings of greatness and power [33].

Family factors are placed on the interpersonal level and affect individual behaviors through two groups of issues: relationship between family and adolescents, and family conditions and atmosphere. The probability of the incidence of such behaviors increases in parents having autocratic parenting styles and negligent parenting style. Similarly, the study by Moussa showed the significant relationship between parenting style and high-risk behaviors [34]. ‘Family conflicts’ is another risk factor. The main impact of the conflicts is unfriendliness between parents and their children. Nelson et al. revealed that there is a relationship between parental conflicts, use of marijuana, and having sexual intercourse by adolescents [35]. Baferani also mentioned that low self-esteem and self-confidence in adolescents are influenced by autocratic parenting style and family conflict [36].

The findings showed that the financial support of adolescents by their families regardless of family income is a risk factor. The likelihood of high-risk behaviors in adolescents may increase due to increase in teenage pocket money. A study by Oljira et al. in eastern Ethiopia indicated that premarital sex was more prevalent among the adolescents who received more pocket money [37].

The findings also showed that the prevalence of these behaviors was higher in families with a family history of antisocial behaviors or a positive attitude toward such behaviors. Kingston’s study indicated that drug and alcohol consumption was higher in adolescents whose parents had a positive attitude toward using them or provided the situation for drug and alcohol consumption [38].

Another identified factor is family income. In this regard, findings of the studies suggested contradictory results. Two studies referred to family income, one of which considered high-income a cause of the occurrence. Siyan Yi et al. also referred to the positive relationship between high family income and sexual behavior in high school students in Cambodia [39]^.^ Another study considered low-income as a cause of the occurrence. It should be pointed out that since the import, production, and sale of synthetic drugs and alcohol are illegal in Iran, there is an underground market for such products, and some people produce them at home with low prices; therefore, these substances can be found at a low price. In addition, low family income and its associated challenges are stressors in adolescents that can affect their behaviors [40].

Access to satellites was another factor for the occurrence of such behaviors. Buying, selling and having a satellite are considered crime in Iran. However, access to satellites is possible via the black market, and some families buy satellites in those markets. Satellites cause adolescents to encounter contents inappropriate for their age group [41].

The increase in mother’s employment was another identified factor. In the past, most women were housewives in Iran and they were responsible for housework. Today, due to evolution of the value system, having job has become a value for women. As the social roles of women in Iran change, other socialization institutions such as the education departments responsible for education have failed to adopt the functions of families. Therefore, mother employment may cause to spend less time with adolescents, and poor performance of socialization institutions may increase adolescents’ vulnerability [42]. Another harm caused by maternal employment is an insecure attachment style. Adolescents with an insecure attachment style tend to consume drugs and alcohol to prevent being rejected and achieve a sense of security, or may have sex to keep their partner in an emotional relationship [43].

The number of siblings is another identified factor. Evidence has shown that the fertility rate in Iran has declined over recent years [44]; therefore, the number of adolescents’ siblings has decreased. This has increased the role of friends in teenagers’ lives so that the likelihood of participation is more in friendly parties, where the occurrence of such behaviors is more probable [45].

The absence of both parents in adolescent life was a risk factor. Given lack of jobs in Iran, many fathers are forced to leave their cities and their families to seek jobs in other cities. Moreover, over the last decade, the rate of divorce in Iran has increased [46]; therefore, the absence of both parents can be an important factor in the context of Iran.

Friends factors are also placed on the interpersonal level and affect individual behaviors. Adolescents follow their friends. If these friends are involved with high-risk behaviors, the possibility of the occurrence of these behaviors will increase through following friends and peer pressure. If there are problems in family structure and there is an insecure attachment style, the adolescents’ tendency to friends will increase [47]. Van de Bongardt et al. referred to the impact of friends’ sexual behaviors on adolescents’ sexual practices [47]. Kohli et al. described peer pressure as a risk factor for the occurrence of these behaviors [48].

Schools affect high-risk behaviors through the status of adolescents at school and the overall school structure. Academic failure is an item of teen status at schools which can lead to a change in the group of his/her friends to the ones performing poorly in their education and are often engaged in high-risk behaviors. They may affect the teenager through peer-to-peer phenomenon. The study by Rodrigues et al. showed a positive relationship between academic failure and sexual behaviors and drug abuse [49].

However, various studies have shown the negative effects of drug abuse on academic performance [50]. In fact, misuse of drugs with the aim of improving academic performance is another reason for the occurrence of the behaviors, which was observed mainly among pre-university students. Pre-university level is the last stage of the study in Iranian high schools. After passing this stage, the students take the university entrance exam named Konkoor in Iran [51].

In this regard, the structure of education and schools in Iran, contrary to its mission (being active both in the fields of educating and breeding students), had a very poor performance in response to the adolescents’ social needs [52].

Another influential factor is a social disorder from community factor theme. Iran, as a developing country, was a traditional society with high social cohesion. As the Iranian society moved toward modernity, social capital decreased. Social control and social cohesion became very poor. The society became confused due to the onset of conflicting values. Thus, social control as an obstacle to the occurrence of high-risk behaviors no longer exists as in the past [53].

Another issue is the lack of appropriate recreation. In Iran, there are a few recreational activities for teens to spend their leisure time. These activities are mainly modern recreational ones, such as going to computer gaming centers, which increase the likelihood of engaging in high-risk behaviors through becoming familiar with peers involved in such behaviors. Additionally, most of these activities are very expensive and inaccessible to all teens. Therefore, due to easy access to drugs and alcohol at low prices, they can be used as a recreation [54]. In their research, Chiapetti et al. concluded that one of the reasons for drug abuse was its fun [55]. Weybright also referred to the protective role of leisure activities against drug abuse [56].

On the other hand, there is the issue of Internet access. Internet has been provided in Iran over a few last years. Although filtering is active in Iran, through the use of filter breakers or VPNs (which are easy to access), the Internet has made it easier for adolescents to have access to sexual contents and interact with their opposite gender through virtual networks [57]. Donevan and Mattebo’s study showed that the desire to have sex and increase sexual partners was higher in adolescents who watch porn movies [58].

## Conclusion

The set of individual, family, friends, school and community factors affect occurrence of these behaviors. Given the identified risk factors, policy makers can design interventions based on identified factors to prevent these behaviors. Among the identified factors most sub-themes belong to family factors; since family is the closest environment that can support adolescents, the role of family is very vital. Many of the identified factors rooted in shortcomings in the family. For example, problems in family structure increase the tendency toward friends.

Iran is a country with ideology of Islam; however, being Muslim does not guarantee adherence to all Islamic guidelines. So being Muslim is not a good reason to prevent these behaviors. Iran needs precise policy making in this area. Therefore, although various factors influence high-risk behaviors in adolescents, and reducing such behaviors in this age group requires policy making and designing systematic interventions at all identified levels, family structure needed to be taken into special consideration. In this regard, some policies should be made in this area based on the healthy family approach. It is also suggested that more primary studies concerning the political, economic, or policy factors affecting such behaviors should be conducted.

### Strengths and weaknesses

We gathered all the relevant studies, regardless of their research methodologies, to provide a narrative review of these behaviors. The findings of this study can be used for other countries with similar conditions. However, we were not able to determine the severity and weakness of these factors. In the reviewed studies, no primary study referred to the political, economic, or policy factors affecting such behaviors.

## Data Availability

The datasets used and/or analyzed during the current study are available from the corresponding author on reasonable request.
